# Neuroprotective Effects of Bacterial Melanin in a Rotenone-Induced Parkinson’s Disease Rat Model: Electrophysiological Evidence from Cortical Stimulation of Substantia Nigra Neurons

**DOI:** 10.3390/biomedicines13061317

**Published:** 2025-05-28

**Authors:** John Sarkissian, Michael Poghosyan, Margarita Danielyan, Narek Makaryan, Tigran Petrosyan, Sona Avetisyan, Anichka Hovsepyan

**Affiliations:** 1 L.A. Orbeli Institute of Physiology NAS RA, Yerevan 0028, Armenia; 2 Synopsys Armenia CJSC, Yerevan 0026, Armenia; 3 Medical Institute, Yerevan Haybusak University, Yerevan 0038, Armenia; 4 Research Center, Armenian State Institute of Physical Culture and Sport, Yerevan 0070, Armenia; 5 SPC “Armbiotechnology” SNPO NAS, RA, Yerevan 0051, Armenia

**Keywords:** Parkinson’s disease model, substantia nigra, rotenone infusion, melanin, electrical activity, cortico-nigral stimulation, modulation of neuronal firing

## Abstract

**Background/Objectives:** As the regulatory center for basal ganglia, the substantia nigra is involved in the pathophysiology of dopaminergic dysregulation in Parkinson’s disease (PD). Increasing neuronal excitability of dopaminergic neurons by different therapeutic methods could reverse the locomotor disturbances of PD. The purpose of this study was the comparative assessment of effects induced by excitatory output from the motor cortex to the substantia nigra (SN) and to investigate the pattern of neuronal responses in an experimental rat model of rotenone-induced (intracerebral infusion) neurodegeneration and treated with bacterial melanin (BM). **Methods:** Thirty-three rats were divided into three groups: control or intact animals (*n* = 12), animals with the rotenone-induced model of PD (*n* = 10), and animals with the PD model and treated with BM in 48 h following the infusion (*n* = 11). Registration of neuronal activity from SN neurons was conducted at four weeks following the rotenone administration. High-frequency stimulation of brain cortical area M1 was performed and the background and evoked activity patterns of 622 neurons were recorded. The difference between the groups was analyzed using one-way ANOVA followed by Tukey’s test. **Results:** A statistically significant difference was observed between the similar proportions of post-stimulus effects registered in different groups, showing the predominance of excitatory responses in the neurons of the melanin-treated group. A comparison of the firing pattern between the SNc and SNr neurons did not reveal significant differences. **Conclusions:** BM treatment has the potential to enhance motor recovery after neurodegeneration in the SN. Deep brain stimulation via the cortico-nigral pathway, with the application of BM, enhances electrical activity in dopaminergic neurons of the substantia nigra and could be a potential therapeutic model for PD.

## 1. Introduction

The substantia nigra (SN) is a regulatory neuronal structure modulating the activity of basal ganglia (BG). The posteromedial region of the SN, known as the pars compacta (SNc), is one of the four brain dopaminergic (DA) nuclei clusters [1], mainly connected with dorsal striatum [2]. The antero-lateral zone of the SN is presented by pars reticularis (SNr) and consists of GABAergic neurons that perceive afferent input from striatum and subthalamic nuclei, projecting it to the ventral anterior thalamic nucleus [3]. The subcortical network of the SNc and SNr has been meticulously studied and described by different authors [4,5,6,7,8,9,10].

Excitatory input from the cerebral cortex reaches the striatum and transfers to the SNr through direct and indirect pathways. The indirect pathway involves the external segment of the globus pallidus and the subthalamic nucleus [5]. Maurice and co-authors have described the organization of the rat prefrontal tract originating from the prelimbic and medial orbital zones and providing excitatory input to the ventral striatum, which projects to the SNr [6,7].

Cortico-nigral projections have been documented in humans as well. Magnetic resonance imaging has provided the mapping of cortical projections to the SNc and SNr involving the thalamus. Visualization methods have revealed links between the SNc and prefrontal cortex (PFC) and SNr connections with the motor and premotor cortex [9]. The neurohistology has described the whole pattern of SN connections with different structures of the brain (corpus callosum, primary sensory cortex, premotor cortex, nucleus caudatus, putamen, nucleus accumbens, temporal occipital lobes, pontine basis, anterior lobe of the cerebellum, and external capsule) [11]. Research evidence has shown that postsynaptic potentials in pars compacta neurons are evoked exclusively from a specific region of stimulation within the pars reticulata, so cortico-nigral stimuli to the SNr also affect the SNc [12]. Physiological effects of cortico-nigral stimulation depend significantly on which part of the SN is engaged due to differences in connectivity and which cortical area is stimulated. Stimulation of the medial prefrontal cortex or primary motor cortex will differentially engage the SNc/SNr. M1 stimulation is primarily motor-related and enhances movements by modulating motor circuits, whereas medial prefrontal cortex stimulation is more related to cognitive and emotional regulation, influencing motivation and reward systems via the SNc [13]. Excitatory inputs cause different responses in the SNc and SNr because of the different neurotransmitters, receptors, and circuit roles in these regions. The SNc uses dopaminergic modulation to influence movement and reward, while the SNr uses GABAergic inhibition to control motor output. These functional differences shape how each region processes excitatory input and ultimately influences motor behavior [13].

As the regulatory center for BG [14], the SN is involved in the pathophysiology of various neurological and neuropsychiatric diseases. Among these conditions, Parkinson’s disease (PD), schizophrenia, pathological inclinations, and addictions are the most extensively studied entities [15,16,17]. Mechanisms of DA dysregulation in the mesencephalon are the core processes in the pathogenesis of Parkinson’s disease, the most common pathology with SN dysfunction [18].

The mechanism of Parkinson’s disease leads to a decreased firing rate of the SN neurons, manifesting also with bursting spikes of registered impulses [19].

Disturbances in electrical processes in cortical connections with the SN in humans include the PFC, pre- and post-central gyrus, and the superior parietal lobe, which are involved in the basic pathophysiological events typical of PD, schizophrenia, and addiction [20]. Research evidence from animal and human studies confirms the hypothesis that the SN is not only part of the BG subcortical network but is functionally also related to the cortex through an additional cortico-nigral pathway identified in humans [21]. The above-mentioned tractographic study in humans revealed a greater proportion of links between the motor or premotor cortex and the SNr compared to the SNc [22].

Increasing neuronal excitability of dopaminergic neurons by different therapeutic methods could reverse the locomotor disturbances in PD. The melanin used in this study was obtained at the Armenian Institute of Biotechnology [23] and was extensively tested in various animal and in vitro models [23,24,25,26,27,28,29,30,31]. In this study, the influence of bacterial melanin was used to analyze the possibilities of modulating SN neuronal activity in a rotenone-induced neurodegeneration model and in response to cortico-nigral stimulation. Melanin is a powerful antioxidant that has an estimated anti-inflammatory action in the brain [30]. BM crosses the blood–brain barrier and can potentially increase the electrical activity of cortical neurons, synchronously stimulating the neurons of the SN region [27,32].

Based on a therapeutic strategy of increasing the activity of neurons along the basal ganglia–thalamus circuitry, deep brain stimulation (DBS) has been implemented as an alternative approach to pharmacotherapy [33].

Dopaminergic neurons are particularly vulnerable to oxidative damage due to the dopamine metabolism itself producing reactive oxygen species (ROS); high mitochondrial activity in neurons, which can leak ROS; and iron accumulation in the substantia nigra, which catalyzes Fenton reactions, generating highly toxic hydroxyl radicals. ROS damages cellular components, ultimately impairing mitochondrial function and triggering apoptosis. Microglial activation is a major hallmark in PD. Once activated, microglia release pro-inflammatory cytokines, which further contribute to oxidative stress. Chronic inflammation sustains a vicious cycle of neurodegeneration, where degenerating neurons stimulate more microglial activation, worsening the damage. Bacterial melanin can neutralize free radicals, reducing oxidative damage to neurons. By reducing oxidative stress, it stabilizes mitochondrial membrane potential and prevents cytochrome c release, curbing mitochondrial-mediated apoptosis. BM can bind transition metals, minimizing ROS generation via metal-catalyzed reactions. Existing pharmacotherapy strategies that replenish brain dopamine levels lead to motor fluctuations and dyskinesias in long-term management. In many patients, psychiatric side effects have been reported. Deep brain stimulation modulates dysfunctional neural circuits and reduces motor symptoms, but it is an invasive and expensive method, not suitable for all patients (the elderly or patients with cognitive impairment), and it does not prevent further neurodegeneration. Complementary benefits of agents like bacterial melanin can address oxidative stress and neuroinflammation, providing additional neuroprotection that DBS cannot [34]. Future studies incorporating electrophysiological assessments, neurochemical profiling, and receptor binding analyses would be essential to clarify how BM affects neurotransmitter release, receptor expression, and the synaptic architecture of the SN and its connected motor pathways. Such insights could help position bacterial melanin as a candidate for adjunctive neurorestorative therapies in Parkinson’s disease and other movement disorders.

The purpose of this study was to conduct a comparative assessment of effects in response to induced excitatory output from the motor cortex to the SN and to investigate the pattern of neuronal responses in rats with rotenone-induced neurodegeneration and treated with bacterial melanin. The comparative functional activity of cortico-nigral connections was assessed with micro-electrophysiological investigation, analyzing the ratio of excitatory and inhibitory processes in neurons of the SNc and SNr in response to induced activity from the primary motor cortex.

## 2. Materials and Methods

### 2.1. Experimental Animals

Thirty-six albino male rats (230 ± 30 g) were used in the electrophysiological study. The animals underwent seven days of acclimation in the same facility (at a temperature of 22 ± 2 °C and 50 ± 15% humidity). All rats received only a standard commercial diet. The animals were randomly divided into three groups: the control group, including intact animals (*n* = 12); the PD model group, including animals with the rotenone-induced model of PD (*n* = 12); and the BM-treated group, including animals with the PD model and treated with melanin (*n* = 12). Following the rotenone infusion, two animals died from the group with the rotenone-induced model of PD (*n* = 10), and another rat was lost in the PD group treated with BM (*n* = 11). The sample size was determined based on precedent from similar preclinical neuropharmacological studies, aiming to balance ethical considerations in animal research with the need for statistical rigor. An a priori power analysis was conducted to estimate the minimum number of animals required to achieve adequate statistical power (80% or higher) at a predefined significance level of α = 0.05.

### 2.2. Rotenone Infusion and Injection of Melanin

The rotenone administration, following the pentobarbital anesthesia (40 mg/kg, i/p), was performed at the rate of 12 μg in 0.5 μL dimexide (1 μL/min) into the “medial forebrain bundle” according to coordinates of the stereotaxic atlas (AP +0.2; L ±1.8; DV +8 mm) [35]. Stereotaxic apparatus-based trepanation of the skull was performed in anesthetized animals (urethane 1.5 g/kg, i/p) from bregma to lambda, dissecting the dura mater. Rotenone solution was injected at a flow rate of 0.2 mL/min, and the needle was additionally left in place for five minutes to ensure complete diffusion of the drug [35]. After the infusion, all animals received proper postoperative care until they recovered completely. Intraperitoneal one-time injection of bacterial melanin was performed at a rate of 170 mg/kg, intraperitoneally, in 48 h following the rotenone infusion. The selection of a 12 µg dose of rotenone for inducing PD pathology in animal models is grounded in its ability to replicate key features of the disease, including dopaminergic neuron degeneration and α-synuclein aggregation. This dose has been employed in various studies to establish reproducible and quantifiable models of PD [36].

Following the completion of all experimental procedures, animals were monitored closely to assess their health status and identify any signs of pain, distress, or abnormal neurological function. Observations were conducted at regular intervals (e.g., every 4–6 h for the first 24 h post-procedure, and twice daily thereafter) by trained personnel in accordance with institutional animal care and use guidelines. Monitoring parameters included body weight, food and water intake, grooming behavior, posture and mobility, response to stimuli, and signs of neurological deficits (e.g., seizures, tremors, or paralysis). In cases where animals exhibited severe clinical signs or reached predefined humane endpoints—such as persistent weight loss (>20%), immobility, labored breathing, self-mutilation, or irreversible neurological impairment—euthanasia was performed immediately to prevent unnecessary suffering. Euthanasia was carried out using an overdose of a barbiturate agent, specifically sodium pentobarbital, administered intraperitoneally at a dosage of ≥150–200 mg/kg, consistent with AVMA Guidelines for the Euthanasia of Animals (2020). The choice of intraperitoneal injection was based on its widespread use and reliability in rodents when intravenous access is impractical.

### 2.3. Electrophysiological Registration

**Figure 1:**
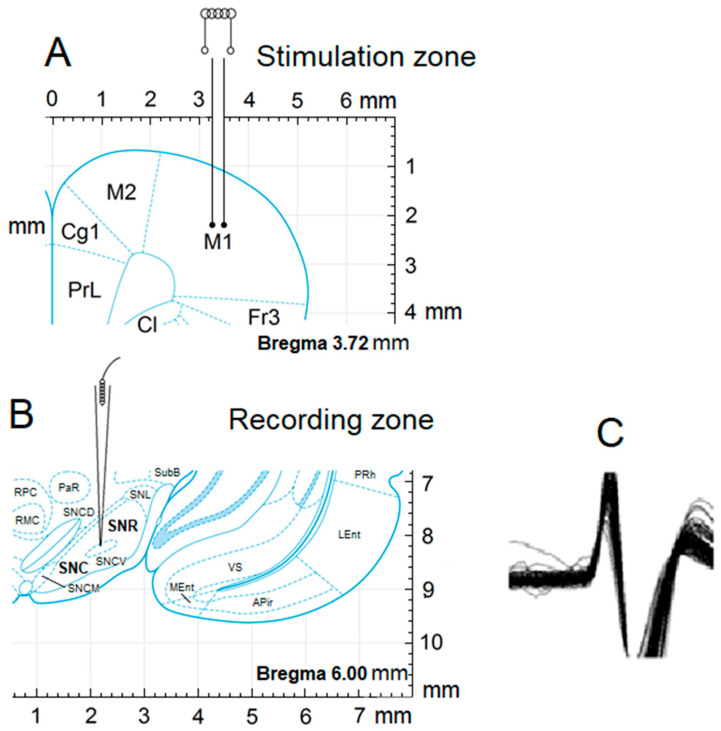
Scheme of experiments by stimulation of M1 and registering from the SNc and SNr. Stereotactic image of the point of neuronal activity recording (**A**), stimulation zones (**B**), and the pattern of action potential (**C**).

Registration of neuronal activity from SN neurons was conducted four weeks following the rotenone infusion. After craniotomy, glass microelectrodes with a tip diameter of 1–2 µM and prefilled with 2M NaCl were inserted in the SNc (AP −5.0; L ±2.0; DV +8.1 mm) and SNr (AP −5.1, L ±2.0, DV +8.6 mm) for extracellular recording of neuronal spike activity. A stimulating tungsten electrode was implanted in ipsilateral M1 (primary motor cortex, Figure 1) according to stereotaxic coordinates (AP +2.1, L ±2.6, DV +1.6 mm). High-frequency stimulation (HFS) of M1 was performed with rectangular current pulses (duration −0.05 ms, with an amplitude of 0.12–0.18 mV, current strength 0.32 mA, and frequency of 100 Hz during 1 s). Dopaminergic neurons in the SN, particularly in the substantia nigra pars compacta (SNc), exhibit two main firing patterns: tonic (~1–8 Hz) and phasic or burst firing, which can reach 80–100 Hz. Burst firing is behaviorally relevant, often occurring during reward-related activity or novel stimuli. Mimicking this pattern with 100 Hz stimulation helps simulate natural high-frequency neuronal activity and dopaminergic signaling. Guehl et al. (2003) demonstrated that 100 Hz stimulation of SN modulated cortical oscillations and motor function in rats [37].

The activity patterns of 622 neurons were recorded. Background activity was registered from neurons before the HFS, and the post-tetanic (stimulation) effects were in the form of post-tetanic depression (PTD) and potentiation (PTP). Tetanic depression (TD) refers to a decrease in the activity (firing rate) of neurons in response to high-frequency electrical stimulation. Tetanic potentiation (TP), in contrast, refers to an increase in neuronal activity during the same type of stimulation, when neurons become more active or “excited” in response to the stimulation. These changes in neuronal firing patterns reflect how brain cells adapt to intense stimulation. These patterns are electrophysiological, but they are associated with changes in movement or behavior. TD/PTD and TP/PTP electrical signatures describe how neurons in the substantia nigra pars compacta (SNc) respond to stimulation. The SNc is deeply involved in motor control, learning, and reward. Changes in SNc activity influence dopamine release in target areas like the striatum, which plays a direct role in initiating or modulating movement. TD/PTD patterns might suggest suppression of dopamine output, possibly associated with movement inhibition or a decrease in motivation or motor readiness. TP/PTP patterns could reflect enhanced dopaminergic activity, potentially linked to movement facilitation, reward anticipation, or increased alertness. In an online mode, a software mathematical analysis of the spiking activity of neurons was conducted. The software used (developed at the Institute of Physiology by a group of software engineers) constructs complex averaged and summed peri-event time histograms (PETH) based on the number of spikes, providing a difference curve and a histogram of frequency, with the calculated average frequency of spikes [32]. The software program allows selection of spikes by amplitude discrimination, withdrawing the “raster” of peristimular spiking, building summarized histograms and diagrams of average spiking frequency with processing of pre- and post-stimulus segments. For selected comparison groups of registered neurons, summed and averaged PETH and frequency histograms were built. An analysis of the obtained data was performed based on a developed software algorithm, providing the precise pattern of peri-stimulus inter-spike intervals. The homogeneity of two independent samples was assessed using Student’s *t-*criterion. A comparative analysis of the critical values in normal distribution was performed at a significance level of 0.05.

A comparative analysis of the impulse activity of single neurons of the SNc (107 neurons, *n* = 5) and SNr (135 neurons, *n* = 7) following the HFS of M1 in the control group, in the PD model (105 SNc neurons, *n* = 5; 184 SNr neurons, *n* = 5) and in melanin-injected animals with the rotenone-induced PD model (95 SNc neurons, *n* = 6; 141 SNr neurons, *n* = 5, respectively), was conducted.

### 2.4. Ethical Information

The study was conducted in accordance with the Declaration of Helsinki, approved by the local institutional ethical committee. All procedures followed national and international regulations on animal experimental use and were approved by the local ethical committee of SPC “Armbiotechnology” SNPO NAS.

### 2.5. Statistical Analysis

The data registered in the electrophysiological study were presented as the mean and standard deviation (mean ± SD). Prior to applying parametric statistical tests, the data were screened for normality and homogeneity of variance. Normality was assessed via the Shapiro–Wilk test, and Levene’s test was used to evaluate the equality of variances across groups. In addition to *p*-values, effect sizes were reported to provide a measure of the magnitude of observed differences. Cohen’s *d* was calculated for pairwise comparisons, with values of 0.2, 0.5, and 0.8 interpreted as small, medium, and large effects, respectively. The difference between the groups was analyzed using one-way analysis of variance (ANOVA), followed by Tukey’s test. For the statistical analyses, SPSS software (version 28.0, IBM Corp., Armonk, NY, USA) was used. A *p*-value of less than 0.05 was considered statistically significant.

## 3. Results

**Figure 2:**
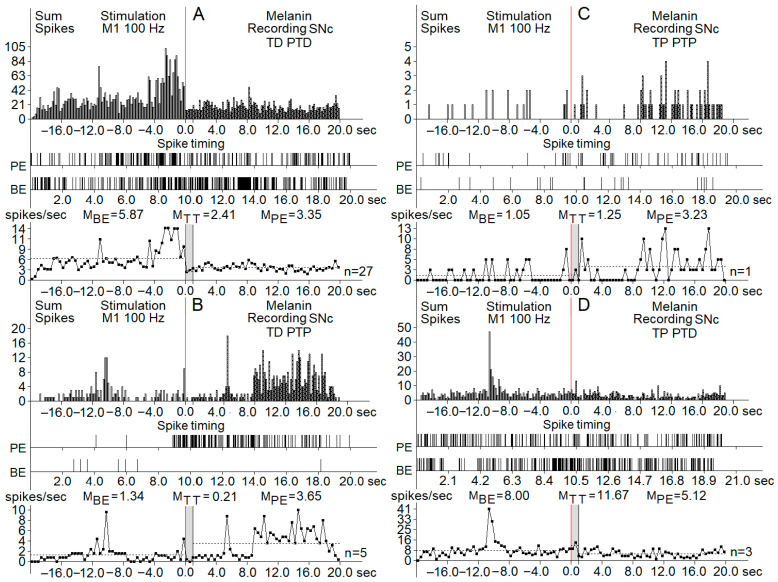
Real-time histograms of neuronal spike activity recorded from melanin-containing neurons in the substantia nigra pars compacta (SNc) during and around high-frequency stimulation (HFS) of the primary motor cortex (M1) at 100 Hz. Each panel (**A**–**D**) shows distinct neuronal response patterns categorized by changes in firing rate: (**A**) TD PTD (tetanic depression with post-tetanic depression): neuronal activity decreases both during and after stimulation. (**B**) TD PTP (tetanic depression with post-tetanic potentiation): firing decreases during stimulation but increases afterward. (C) TP PTP (tetanic potentiation with oost-tetanic potentiation): firing increases during stimulation and remains elevated afterward. (**D**) TP PTD (tetanic potentiation with post-tetanic depression): firing increases during stimulation and decreases after it ends. Visual breakdown (applies to all panels (**A**–**D**)): (top row) (sum spikes): histograms of total spike counts across time, aligned around the stimulation onset at 0 s (red vertical line). Data span from 20 s before to 20 s after stimulation. (middle row) (spike timing raster): raster plots showing spike timing of individual neurons, providing a visual of the firing patterns across trials. (bottom row) (spike frequency plot): averaged firing rates across three time windows: BE (before event)—baseline period, TT (tetanization time)—during stimulation, PE (post-event)—after stimulation. Spike rates (in spikes/s) are annotated as mean values for each time window (M_BE_, M_TT_, M_PE_). The number of neurons tested per pattern (n) is listed to the right of each frequency plot.

**Figure 3:**
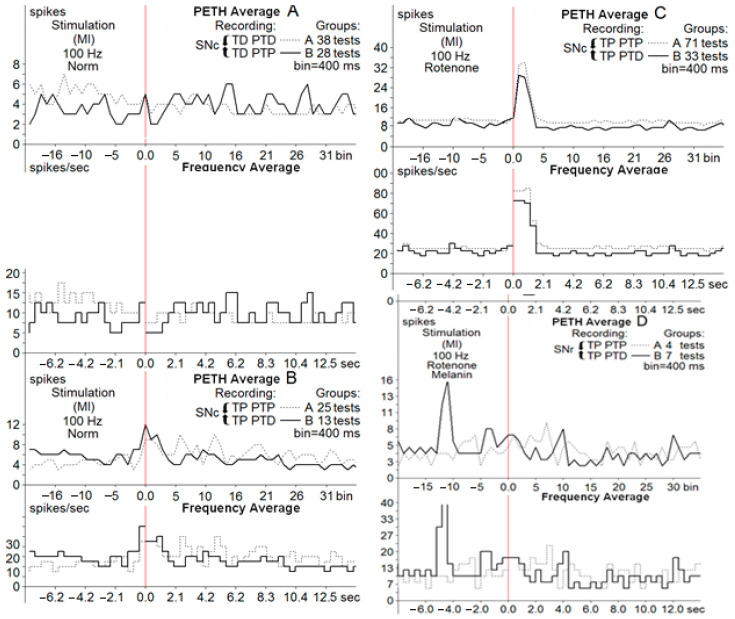
Average peri-event time histograms (PETH average) and frequency histograms (frequency average) of depressor, depressor excitatory ((**A**), Groups A, B) and excitatory, excitatory depressor ((**B**,**C**), Groups A, B) post-stimulus manifestations of SNc neurons at HFS (100 Hz, 1 s) of M1 in norm ((**A**,**B**), Groups A, B), in neurons of the PD model (**C**) and in neurons of melanin-treated rats (**D**).

Post-stimulation analysis was conducted based on the estimated average pulse intervals and frequencies, comparing results with the pre-stimulus levels, specifying the inhibitory and excitatory tetanic (peri-stimulus tetanic depression (TD) or potentiation (TP)) and post-tetanic reactions (TD followed by PTD (post-tetanic depression)), TD followed by PTP (post-tetanic potentiation), TP followed by PTP, and TP followed by PTD. In the registered diagrams, the tetanic potentiation (TP) is manifested as an increase in the spiking amplitude of neurons and tetanic depression in the form of suppressed spiking activity with decreased amplitude (Figure 2 and Figure 3). Similarly, PTP and PTD were evidenced by the increased (PTP) or decreased spiking amplitude and rate.

**Figure 4:**
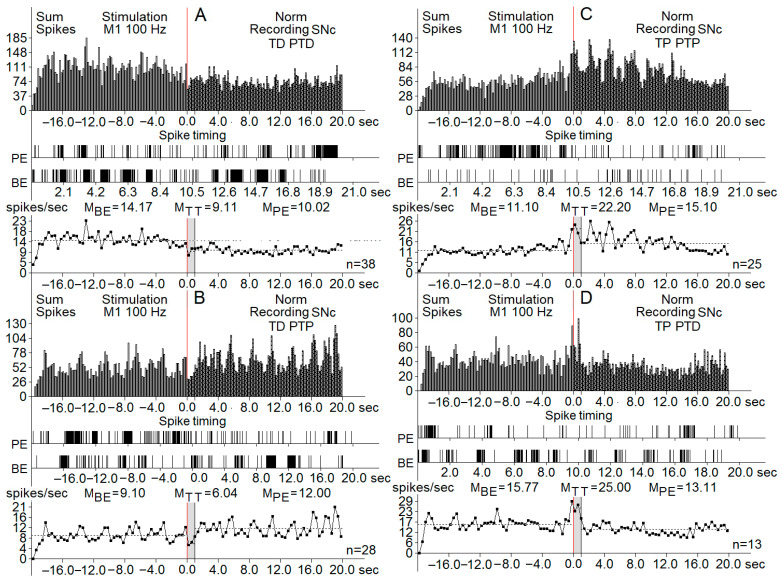
Real-time summed histograms of spike activity from substantia nigra pars compacta (SNc) neurons (*n* = 622 total), recorded before, during, and after high-frequency stimulation (HFS) of the primary motor cortex (M1) at 100 Hz. Each panel (**A**–**D**) represents different manifestations of neuronal response patterns: (**A**) TD-PTD (tetanic depression with post-tetanic depression): displays a net reduction in spike activity both during and after stimulation. (**B**) TD-PTP (tetanic depression with post-tetanic potentiation): characterized by suppressed activity during stimulation followed by increased activity afterward. (**C**) TP PTP (tetanic potentiation with post-tetanic potentiation): shows enhanced neuronal firing both during and after the tetanic stimulus. (**D**) TP PTD (tetanic potentiation with post-tetanic depression): displays excitation during the stimulus and suppression post-stimulation. Each panel includes: (top row) (sum spikes): summed histogram showing spike counts over time (±20 s around the stimulation event at time 0, indicated by the red vertical line). (middle row) (spike timing raster): raster plots of spike timings from representative individual neurons. (bottom row) (frequency histograms): plots showing average firing frequency across three time windows: BE (before event)—baseline period, TT (tetanization time)—during stimulation, PE (post-event)—after stimulation. Mean values of spike rates (M_BE_, M_TT_, M_PE_) are annotated above each frequency graph, and the number of recorded trials (n) is shown to the right of each panel. The grey area in the image marked MTT is the stimulation period.

The pre-stimulation firing rate was recorded in three clusters of neurons (control, PD model, and PD model with melanin injections). The background firing rates in the neurons of the three groups before the HFS (before-event-M_BE_) were registered as synaptic excitatory responses or inhibitory synaptic responses (Figure 4).

Registration of the post-stimulation activity of neurons showed different patterns of responses in the three groups of SNc and SNr neurons. The effects of cortical HFS are classified as peri-stimulation and post-stimulation responses. Tetanic depression (TD) or tetanic potentiation (TP) was recorded during the stimulation process (peri-stimulation changes). Post-stimulation effects had the same patterns: post-tetanic depression (PTD) and post-tetanic potentiation (PTP). The overall types of post-stimulatory responses were in the form of TD-PTD, TD-PTP, TP-PTD, and TP-PTP. Figure 2 and Figure 3 present samples of registration from different groups of neurons.

Another parameter used for the description of post-stimulus effects was the spiking frequency. All types of excitatory post-effects were associated with a higher spiking frequency, whereas in inhibitory post-stimulus responses, the frequency was similar to the pre-stimulus rate or lower. An analysis of the firing rate using the frequency curves is shown in Figure 4, including the changes in spiking activity of neurons of the PD model group.

Proportions of post-stimulus effects were derived for the neurons of the SNc and SNr in different groups to show the difference in responses between the SNc and SNr neurons (Table 1). Similarly, the ratio of initial and post-stimulus excitatory and inhibitory effects was derived to show clearly the pattern of responses in different groups and for different clusters of neurons (Table 2).

A ratio analysis of neuronal response effects was performed to reveal the preponderance of excitatory or inhibitory patterns in response to tetanic stimulation. The ratio of excitatory and inhibitory effects registered in three clusters of neurons was measured to compare the pre-stimulation firing patterns (before event M_BE_) with effects registered during the stimulation (M_TT_) and after stimulation (post-event M_PE_).

A statistically significant difference was observed between the similar proportions of post-stimulus effects (rotenone-injected group vs. BM-treated group), showing the predominance of excitatory responses in neurons of the melanin-treated group (Table 2). The BM-treated group scored significantly higher than the control group on the post-test, *p* < 0.05, Cohen’s *d* = 0.90, indicating a significant effect. A comparison of firing patterns between the SNc and SNr neurons (*p* = 0.24, Cohen’s *d* = 0.36) did not reveal a significant difference (Table 1 and Table 2).

## 4. Discussion

BM significantly increased the firing rate of SNc dopaminergic neurons and enhanced excitatory responses following high-frequency stimulation of the caudate–putamen. These findings suggested that BM may facilitate recovery and regeneration processes after central nervous system lesions by modulating neuronal activity in the SNc. Effects of BM on neuronal activity were also evaluated in the rat sensorimotor cortex [28,33]. BM application led to increased excitability and facilitated post-stimulation potentiation, indicating that BM’s neuromodulatory effects are not limited to the SNc but extend to other brain regions involved in motor control and learning. In the presented study, BM was used to evaluate the effects of bacterial melanin in a rat model of Parkinson’s disease induced by rotenone administration with the purpose of revealing potential neuroprotective effects. Bacterial melanin is characterized by its high solubility, biocompatibility, and antioxidant capacity, which make it a particularly attractive candidate for therapeutic application in neurodegenerative and neurotraumatic conditions. Its protective mechanisms appear to act across multiple pathological domains, including oxidative stress, neuroinflammation, and neuronal excitability, all of which are central to the progression of neurological diseases. BM exhibits strong antioxidant activity, neutralizing reactive oxygen species such as superoxide anions, hydroxyl radicals, and hydrogen peroxide. This activity protects lipids, proteins, and DNA from oxidative damage. BM may help preserve mitochondrial function by limiting oxidative stress within the organelle, thereby sustaining ATP production and reducing the release of pro-apoptotic factors.

The SN is not only a part of the subcortical basal ganglia network, but it is also connected with the cortex by means of an additional, parallel circuit. Menke et al. used probabilistic DTI tractography with the aim of differentiating the SNc and SNr according to their different connectivity profile. The SNc showed a higher connectivity profile with the prefrontal cortex, whilst the SNr was more connected with motor and premotor cortices [9]. However, dopaminergic neurons of the SNc are highly connected with the SN pars reticulata, a major output nucleus of the basal ganglia. These connections explain the similar ratios of pattern responses registered in neurons of both structures in our study. The postsynaptic potentials in pars compacta neurons are evoked exclusively from a specific region of stimulation within the pars reticulata, so cortico-nigral stimuli to the SNr also affect the SNc. High cross-correlation between SNc and SNr firing suggests the preserved integrity of intranigral connections in BM-treated animals.

One of the evidence-based methods used for the treatment of Parkinson’s disease is deep brain stimulation. The method is a surgical intervention used to treat mostly the movement symptoms of PD. The main approved brain targets used in such PD treatment are the subthalamic nucleus and the globus pallidus interna. The target choice largely depends on individual needs. There are many ongoing studies that will help to refine target choice for individual people (including the cortical pathway). While the open-loop stimulation of deep brain structures (subthalamic nucleus and internal part of the globus pallidus) remains the main approach in therapies for PD, the possible therapeutic use of stimulation to superficial cortical areas has been studied by some research groups [38,39].

Still, in 1979, Woolsey et al. reported that stimulation of the cortical M1 region with subthreshold intensities leads to a temporary improvement in patients with PD [40]. Currently, stimulation of the motor cortex (SMC) is used for different purposes (mostly pain-associated motor disorders) [41,42]. SMC is performed using epidurally implanted electrodes. However, the clinical efficacy reported by different clinicians and researchers is variable [43]. SMC therapy has shown significant heterogeneity in treatment outcomes. Bentivoglio et al. showed moderate effects of unilateral SMC on axial symptoms and quality of life in PD patients [44].

In a review presented by Anne Beuter et al., the authors underlined the significant differences in selection criteria, surgical procedures, stimulation settings, frequency of stimulation, and methods to assess motor improvement in studies included in their analysis [39].

The mechanisms of action in SMC encompass the activation of hypoactive brain structures and/or inhibition of some hyperactive zones [45]. Our results also confirmed the concept that by stimulating the motor cortex in a PD model, the mechanism of neuromodulatory action consists primarily of firing rate changes in cortical–basal ganglia networks and supposedly results in a modulation of neuronal activity at the cortical level [27].

In our study, the method of direct intra-cerebral infusion was used to generate a rat PD model. This method leads to a progressive loss of dopaminergic neurons in the SN without inducing peripheral toxicity and simultaneously increases the expression of α-synuclein in the brain area [46]. Effects of BM on the electrical activities of substantia nigra dopaminergic neurons have been tested in intra-cerebral infusion model. The preserved neuronal activity in the SN is crucial for PD patients and can prevent motor impairments. The excitability of dopaminergic neurons affects the function of the nigrostriatal system for motor coordination. Neuronal projections of the SNr are output neurons for the basal ganglia and thus critical for movement control. Alterations in neuronal activity lead to a variety of motor disturbances, including resting tremor, rigidity, bradykinesia, and postural instability [47]. In BM-treated animals, a predominance of excitatory effects were registered in response to cortical stimulation, indicating that melanin stimulates the hypoactive areas in the substantia nigra. At the molecular or cellular level, BM may scavenge reactive oxygen species in the SNc, reducing oxidative stress and downregulating caspases. It may influence the synthesis, storage, or degradation of dopamine, protecting neurons from dopamine-related oxidative damage. While SNr neurons are not primarily affected by rotenone, BM might stabilize GABAergic output, possibly through reducing local inflammation, glial activation, or modulating ion channel activity or GABA receptor expression.

Degeneration of dopaminergic neurons within the SNc disrupts the normal balance of excitatory and inhibitory signals across basal ganglia circuits, leading to pathological changes in neuronal firing patterns and a loss of coordinated motor output [48,49]. A growing body of evidence suggests that enhancing excitatory activity in SN neurons, particularly those within the SNc and, under certain conditions, the SNr, may facilitate the restoration of functional circuit dynamics, thereby promoting motor recovery. Therapeutically induced excitatory input to SN neurons has the potential to restore the temporal stability of neuronal firing within the basal ganglia–thalamo-cortical circuit. In particular, increased activity in the SNc may promote phasic dopamine release in the striatum, which plays a critical role in modulating the gain of cortical motor signals by appropriately scaling motor-related output in accordance with task demands [50,51].

Though the four week period used in this study is relatively short-term, potential long-term effects of BM might include increased neuronal survival (continued protection against oxidative damage could slow dopaminergic neuron death), preservation of motor function (If BM sustains SNc-SNr integrity, it might delay motor symptom progression), reduced neuroinflammation (long-term immunomodulatory effects could further preserve surrounding neurons and glia), and improved synaptic plasticity and enhanced neurotransmitter balance could support circuit-level compensation. The administration of BM will delay the disease progression, slowing neuron loss. Additionally, preserved neural circuits might respond better to L-DOPA or deep brain stimulation.

While the present study provides valuable insights into the electrophysiological effects of BM in a rotenone-induced Parkinson’s disease rat model, several important limitations must be acknowledged. The lack of behavioral or motor function assessments limits the ability to directly correlate the observed neuronal activity changes with functional recovery. Without complementary behavioral data—such as assessments of locomotion, coordination, or bradykinesia—it remains unclear whether the electrophysiological improvements translate into meaningful clinical or motor benefits. The study employed a single dose of rotenone, which may not fully capture the variability or progressive nature of PD pathology, potentially limiting the generalizability of the findings. The absence of histological validation, such as immunohistochemical analysis of dopaminergic neuronal survival (e.g., tyrosine hydroxylase staining), glial activation, or oxidative stress markers, precludes a more comprehensive understanding of the underlying cellular and tissue-level changes. Addressing these limitations in future studies will be essential to strengthen the translational relevance of bacterial melanin as a neuroprotective agent in Parkinsonian models. Another limitation in this study was the inability to show the mechanism underlying the firing patterns of neurons from the SNc and SNr regions following BM treatment. the results indicate no significant differences in the firing patterns of neurons from these two regions following BM treatment (*p* = 0.24, Cohen’s *d* = 0.36). The response patterns of SNc and SNr neurons to BM treatment were quite similar, failing to reveal distinct functional differences between these areas. Under typical basal ganglia circuitry, the SNr is a GABAergic inhibitory output nucleus, and the SNc contains dopaminergic neurons that modulate movement and learning. Generally, the SNr exerts inhibitory control over the SNc. However, under certain conditions, stimulation of the SNr can paradoxically result in excitatory activity in the SNc. If stimulation involves SNr interneurons or afferent pathways that inhibit SNr output neurons, the net result can be disinhibition of the SNc, reducing GABAergic inhibition on the SNc and making it excited. Alternatively, SNr stimulation could modulate other nuclei (e.g., the pedunculopontine nucleus, superior colliculus, or thalamus) that send excitatory feedback to the SNc. These polysynaptic loops can result in net excitation of the SNc.

Although the picture is not yet clear on the issue of better target choice for brain stimulation, the use of drugs that increase the electrical activity of SN neurons is an additional method to improve the functionality of SN neurons, when combined with electrical stimulation or without it.

## 5. Conclusions

The present findings demonstrate that bacterial melanin (BM) treatment is associated with an increase in the spiking activity of dopaminergic neurons in the substantia nigra, suggesting a potential modulatory effect on neuronal excitability following neurotoxic injury. These results point to BM as a promising candidate for further exploration in strategies aimed at restoring neural activity within basal ganglia circuits affected by Parkinsonian neurodegeneration. Additionally, the potential synergy between BM administration and stimulation of the cortico-nigral pathway may represent a novel approach for modulating dopaminergic neuron function. However, it is important to emphasize that the current study did not include behavioral or motor assessments, nor histological validation of neuroprotection. Future studies incorporating comprehensive behavioral testing, histopathological analysis, and long-term functional evaluation are essential to substantiate the translational potential of BM in Parkinson’s disease models. New therapeutic strategies might consider combined approaches, integrating neuromodulatory techniques like DBS with pharmacological agents such as bacterial melanin that support cellular resilience and circuit restoration.

## Figures and Tables

**Table 1:** Proportions of effects in response to M1 stimulation.

	SNc Neurons			SNr Neurons		
**Types of Post-Stimulus Effects**	**Control/Intact**	**PD: Rotenone**	**PD: Rotenone and BM**	**Control/Intact**	**PD: Rotenone**	**PD: Rotenone and BM**
TD-PTD	9%	27% *	12% *	13%	32% *	16% *
TD-PTP	36%	20% *	36% *	31%	17% *	39% *
TP-PTP	27%	11% *	25% *	24%	15% *	28% *
TP-PTD	28%	42%	27%	32%	36% *	17% *

* The comparisons where statistically significant differences were observed (*p* < 0.05) between the similar proportions of effects (rotenone-injected group vs. BM-treated group).

**Table 2:** Ratio of excitatory and inhibitory effects registered in three clusters of neurons. The pre-stimulation firing patterns (before-event M_BE_) are compared with registered effects during the stimulation (M_TT_) and after stimulation (post-event M_PE_).

	SNc			SNr		
**Types of Post-Stimulus Effects**	**Control/Intact**	**PD** **: Rotenone**	**PD** **: Rotenone and BM**	**Control/Intact**	**PD-Rotenone**	**PD** **: Rotenone and BM**
Inhibitory (TD-PTD)						
M_BE_/M_TT_	5.36	2.06	4.76	6.04	2.66	5.35
M_BE_/M_PE_	2.88	1.74	1.92	3.23	1.86	2.07
Inhibitory (TD-PTP)						
M_BE_/M_TT_	4.24	2.68	4.41	4.84	3.96	5.22
M_BE_/M_PE_	2.20	2.33	1.98	2.62	3.14	2.58
Excitatory (TP-PTP)						
M_BE_/M_TT_	3.21	2.39	2.87	3.86	2.46	3.72
M_BE_/M_PE_	1.75	1.48	1.80	1.92	2.16	2.23
Excitatory (TP-PTD)						
M_BE_/M_TT_	2.18	5.81	2.08	2.79	6.22	2.78
M_BE_/M_PE_	1.28	4.24	1.12	1.50	4.97	1.42

## Data Availability

The data presented in this study are available upon request from the corresponding author.

## Abbreviations

The following abbreviations are used in this manuscript:

BM	Bacterial melanin
BG	Basal ganglia
DA	Dopaminergic
PD	Parkinson’s disease
SNr	Substantia nigra pars reticulata
SNc	Substantia nigra pars compacta
PFC	Prefrontal cortex
DBS	Deep brain stimulation
AP	Anteroposterior
DV	Dorsoventral
HFS	High-frequency stimulation
PTD	Post-tetanic depression
PTP	Post-tetanic potentiation
PETH	Peri-event time histogram
SPC	Scientific production center
SNPO	State non-profit organization
NAS	National Academy of Science
TP	Tetanic potentiation
TD	Tetanic depression
TT	Time of tetanization
MBE	Measured before the event
MTT	Measured in time of tetanization
MPE	Measured post-event
DTI	Diffusion tensor imaging
SMC	Sensorimotor cortex
